# Down Syndrome: Current Status, Challenges and Future Perspectives

**Published:** 2016-08-10

**Authors:** Mohammad Kazemi, Mansoor Salehi, Majid Kheirollahi

**Affiliations:** 1 *Department of Genetics and Molecular Biology, School of Medicine, Isfahan University of Medical Sciences, Isfahan, Iran.*; 2 *Medical Genetic Center of Genome, Isfahan, Iran.*; 3 *Pediatric Inherited Diseases Research Center, Research Institute for Primordial Prevention of Non-communicable Disease, Isfahan University of Medical Sciences, Isfahan, Iran.*

**Keywords:** Down syndrome, trisomy 21, prenatal diagnosis, chromosome abnormality, cell-free fetal DNA (cffDNA); noninvasive prenatal screening (NIPS)

## Abstract

Down syndrome (DS) is a birth defect with huge medical and social costs, caused by trisomy of whole or part of chromosome 21. It is the most prevalent genetic disease worldwide and the common genetic cause of intellectual disabilities appearing in about 1 in 400-1500 newborns. Although the syndrome had been described thousands of years before, it was named after John Langdon Down who described its clinical description in 1866. Scientists have identified candidate genes that are involved in the formation of specific DS features. These advances in turn may help to develop targeted therapy for persons with trisomy 21. Screening for DS is an important part of routine prenatal care. Until recently, noninvasive screening for aneuploidy depends on the measurement of maternal serum analytes and ultrasonography. More recent progress has resulted in the development of noninvasive prenatal screening (NIPS) test using cell-free fetal DNA sequences isolated from a maternal blood sample. A review on those achievements is discussed.

Down syndrome (DS) is the most frequently occurring chromosomal abnormality in humans and affecting between 1 in 400-1500 babies born in different populations, depending on maternal age, and prenatal screening schedules (1-6). DS is the common genetic cause of intellectual disabilities worldwide and large numbers of patients throughout the world encounter various additional health issues, including heart defects, hematopoietic disorders and early-onset Alzheimer disease (7-9). The syndrome is due to trisomy of the whole or part of chromosome 21 in all or some cells of the body and the subsequent increase in expression due to gene dosage of the trisomic genes (10). It is coupled with mental retardation, congenital heart defects, gastrointestinal anomalies, weak neuromuscular tone, dysmorphic features of the head, neck and airways, audiovestibular and visual impairment, characteristic facial and physical features, hematopoietic disorders and a higher incidence of other medical disorders. The incidence of births of children with DS increases with the age of the mother. However, due to higher fertility rates in younger women, the probability of having a child with DS increases with the age of the mother and more than 80% of children with DS are born to women under 35 years of age (7, 11). 

## Historical background

**Fig 1:**
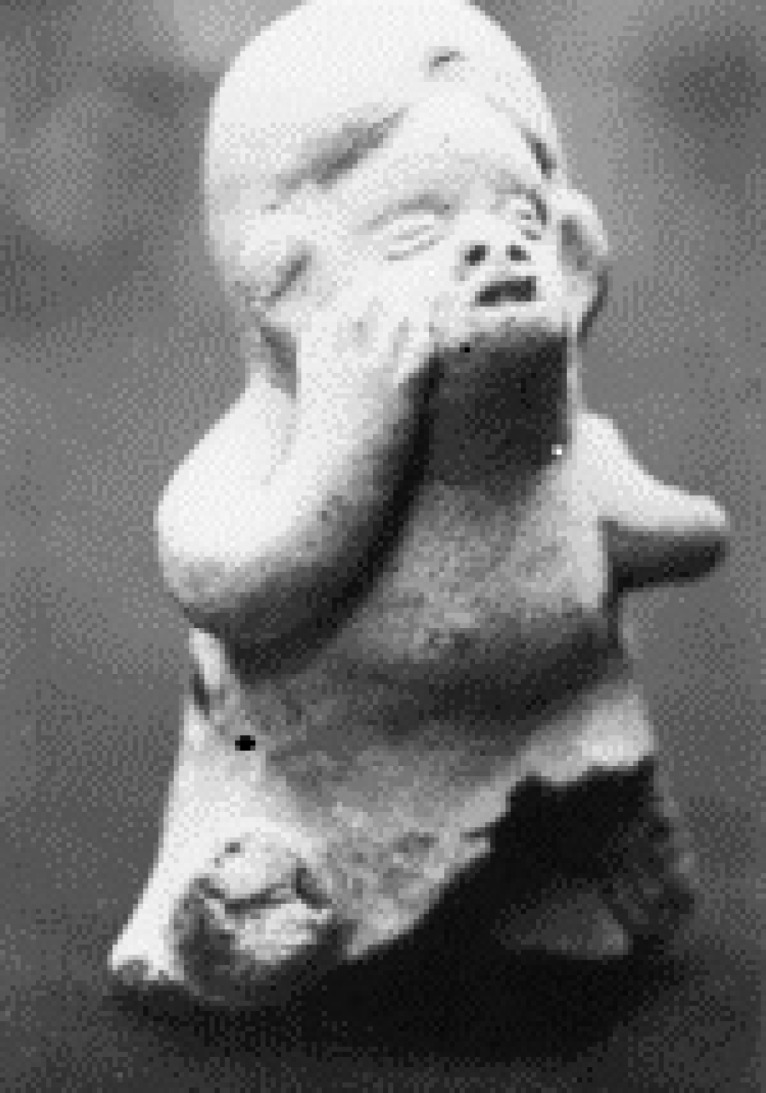
Down syndrome statue representing individual with trisomy 21 related to almost 2500 years ago (16)

Approximately 2500 years ago, Bernal and Briceno thought that certain sculptures represented individuals with trisomy 21, making these potteries the first empirical indication for the existence of the disease (Figure 1). Martinez-Frias identified the syndrome in 500 patients with Alzheimer disease in which the facial features of trisomy 21 are clearly displayed. Different scientists described evident illustration of the syndrome in 15^th^ and 16^th^ century paintings. Esquirol wrote phenotypic description of trisomy 21 in 1838. English physician, John Langdon Down explained the phenotype of children with common features noticeable from other children with mental retardation. He referred them “Mongoloids” because these children looked like people from Mongolia (12-15).

This disease was named “Down Syndrome” in honor of John Langdon Down, the doctor who first recognized the syndrome in 1866 but until the middle of the 20^th^ century, the cause of DS remained unknown. The probability that trisomy 21 might be a result of a chromosomal abnormality was suggested in 1932 by Waardenburg and Davenport (12, 17). A revolution finally took place in 1956, when Joe Hin Tjio and Albert Levan described a set of experimental situations that allowed them to precisely characterize the number of human chromosomes as 46. During the three years of the publication of this revolutionary work, Jerome Lejeune in France and Patricia Jacobs in the United States were able to identify an extra copy of chromosome 21 in karyotypes prepared from DS patients. Then, in the 1959, researchers finally determined that presence of an additional copy of chromosome 21 (referred to trisomy 21) is the cause of DS (1, 18).

**Genetic basis**

Chromosome 21 is the smallest human autosome with 48 million nucleotides and depicts almost 1–1.5% of the human genome. The length of 21q is 33.5 Mb and 21 p is 5–15 Mb. More than 400 genes are estimated to be on chromosome 21 (Table 1). Chromosome 21 has 40.06% repeat content comprising short interspersed repeatitive elements (SINEs), long interspersed repeatitive elements (LINEs), and long terminal repeats (LTRs) (3, 11, 19). The most acceptable theory for the pathogenesis of trisomy 21 is the gene-dosage hypothesis, which declares that all changes are due to the presence of an extra copy of chromosome 21 (12). Although it is difficult to select candidate genes for these phenotypes, data from transgenic mice suggest that only some genes on chromosome 21 may be involved in the phenotypes of DS and some gene products may be more sensitive to gene dosage imbalance than others. These gene products include morphogens, cell adhesion molecules, components of multi-subunit proteins, ligands and their receptors, transcription regulators and transporters. A “critical region” within 21q22 was thought to be responsible for several DS phenotypes including craniofacial abnormalities, congenital heart defects, clinodactyly of the fifth finger, mental retardation and several other features (3, 11).

DS is usually caused by an error in cell division named "nondisjunction" that leads to an embryo with three copies of chromosome 21. This type of DS is called trisomy 21 and accepted to be the major cause of DS, accounting for about 95% of cases (20, 21). Since the late 1950s, scientists have also determined that a smaller number of DS cases (nearly 3-4%) are caused by chromosomal translocations. Because the translocations responsible for DS can be inherited, this form of the disease is sometimes named as familial DS. In these cases, a segment of chromosome 21 is transferred to another chromosome, usually chromosome 14 or 15. When the translocated chromosome with the extra piece of chromosome 21 is inherited together with two common copies of chromosome 21, DS will occur. For couples who have had one child with DS due to translocation trisomy 21, there may be an increased likelihood of DS in future pregnancies. This is because one of the parents may be a balanced carrier of the translocation. The chance of passing the translocation depends on the sex of the parent who carries the rearranged chromosome 21. If the father is the carrier, the risk is around 3 percent, while with the mother as the carrier, the risk is about 12 percent. This difference is due to the fact that it seems to be a selection against chromosomal abnormalities in sperm production which means men would produce fewer sperm with the wrong amount of DNA. Translocation and gonadal mosaicism are types of DS known to have a hereditary component and one third of them (or 1% of all cases of DS) are hereditary (1, 22). The third form of disease named mosaicism, is a rare form (less than 2% of cases) of DS. While similar to simple trisomy 21, the difference is that the third copy of chromosome 21 is present in some, but not all cells. This type of DS is caused by abnormal cell division after fertilization. In cellular mosaicism, the mixture can be seen in different cells of the similar type; while with mosaicism, one set of cells may have normal chromosomes and another type may have trisomy 21 (1, 22)

**Table 1:** Candidate dosage sensitive genes on chromosome 21causing DS phenotype (11, 23, 24)

**Gene Symbol**	**Full Name**	**Location**
APP	amyloid beta (A4) precursor protein	21q21.2|21q21.3
OLIG1	oligodendrocyte transcription factor 1	21q22.11
OLIG2	oligodendrocyte lineage transcription factor 2	21q22.11
DYRK1A	dual-specificity tyrosine-(Y)-phosphorylation regulated kinase 1A	21q22.13
DSCAM	Down syndrome cell adhesion molecule	21q22.2
SYNJ1	synaptojanin 1	21q22.2
JAM2	junctional adhesion molecule 2	21q21.2
SIM2	single-minded homolog 2 (Drosophila)	21q22.2|21q22.13
ERG	v-ets avian erythroblastosis virus E26 oncogene homolog	21q22.3
PTTG1IP	pituitary tumor-transforming 1 interacting protein	21q22.3
ADAMTS1	ADAM metallopeptidase with thrombospondin type 1 motif 1	21q21.3
ITSN1	intersectin 1	21q22.1-q22.2
SYNJ11	synaptojanin 1	21q22.2
ERG	v-ets avian erythroblastosis virus E26 oncogene homolog	21q22.3
ETS2	ETS proto-oncogene 2, transcription factor	21q22.3
SLC19A1	solute carrier family 19 member 1	21q22.3
COL6A1	collagen type VI alpha 1	21q22.3


## Screening methods

Screening for DS is an important part of routine prenatal care. The most common screening method contains the measurement of a combination of factors: advanced maternal age, multiple second trimester serum markers, and second trimester ultrasonography (Table 2) (25-26).

The first method available for aneuploidy screening was maternal age. Advanced maternal age predisposes to DS and other fetal chromosomal abnormalities based on nondisjunction. In fact, the advanced maternal age was defined as age 35 years or older at delivery, because her risk of having a fetus with aneuploidy was equivalent to or more than the estimated risk for pregnancy loss caused by an amniocentesis. The extra chromosome 21 is the result of nondisjunction throughout meiosis in the egg or the sperm (standard trisomy 21) in almost 95% of individuals (27-29).

Trisomy 21 is coupled with a propensity for brachycephaly, duodenal atresia, cardiac defects, mild ventriculomegaly, nasal hypoplasia, echogenic bowel, mild hydronephrosis, shortening of the femur and sandal gap and clinodactyly or middle phalanx hypoplasia of the fifth finger. The first reported marker associated with DS was the thickening of the neck area (30, 31). 40-50 percent of affected fetuses have a thickened nuchal fold measuring ≥ 6 mm in the second-trimester (32, 33). After using of screening by nuchal translucency (NT), about 83% of trisomy 21 pregnancies were identified in the first trimester. Later, it was revealed that screening by a combination of maternal age, NT and bi-test [pregnancy-associated plasma protein (PAPP-A) with second trimester free β chorionic gonadotropin (β-hCG)] or tri-test [alpha-fetoprotein (AFP), estriol and free β-hCG] has a potential sensitivity of 94% for a 5% false-positive rate (34-36).

NT is a physiological process ‘marker’ in the fetus that reflects the fetal lymphatic and vascular development in the head and neck area. NT measurement was primarily used as a stand-alone test for aneuploidy screening. Later, maternal age was added, and finally, NT became part of a combined first trimester aneuploidy screening test (NT, maternal age and the maternal serum markers, PAPP-A and β-hCG) (35).

Pyelectasis which refers to a diameter of the renal pelvis measuring ≥ 4 mm, is another second trimester marker; in fact, renal dilatation has a higher occurrence among fetuses with DS. However, pyelectasis remains a minor marker as the sensitivity is about 17%-25%, with a false-positive rate of 2%-3% (37).

**Table 2:** Detection rates and false positive rates of different Down syndrome screening tests (43, 44)

**Test**	**Markers of aneuploidy**	**Trimester**	**DR (%)**	**FPR (%)**
NT alone	NT	1^st^	64-70	5
Combined	NT+ PAPP-A + β-hCG	1^st^	65	5
Triple screen	β-hCG + AFP + estriol	2^nd^	70	14
Quad screen	β-hCG + AFP + estriol+ inhibinA	2^nd^	81	7
Serum Integrated	β-hCG +AFP +estriol+ inhibinA + PAPP-A	1^st^ and 2^nd^	85-88	5
Integrated	NT + β-hCG + AFP + estriol+ inhibinA + PAPP-A	1^st^ and 2^nd^	94-96	1
Sequential	NT + β-hCG +AFP + estriol+ inhibinA + PAPP-A	1^st^ and 2^nd^	95	2

DR: detection rate; FPR: false-positive rate; NT: nuchal translucency; PAPP-A: pregnancy-associated plasma protein- A; β-hCG: chorionic gonadotropin; AFP: alpha-fetoprotein

Another important soft marker that has been effectively combined into fetal abnormality screening is the nasal bone. The absence of nasal bone in fetus at the 11-14 weeks scan is related to DS. This marker, initially, was found in 73% of trisomy 21 fetuses and in only 0.5% of chromosomally normal fetuses (38, 39) and, subsequently, it was estimated that the combination of maternal age, NT, maternal serum biochemical screening (by bi- test or tri- test) and examination of nasal bone could increase the detection rate to 97% (40). After the completion of further confirmation studies, it is generally accepted that fetal nasal bone is a worthy sonographic marker, even if there are racial differences in the length of this bone (41- 42).

**Noninvasive prenatal screening (NIPS)**

One of the major innovations in obstetrical care was the introduction of prenatal genetic diagnosis, primarily by amniocentesis in the second trimester of pregnancy. Later, chorionic villus sampling during the first trimester allowed for earlier diagnosis. However, the potential risk of fetal loss due to an invasive procedure has urged the search for noninvasive approaches for genetic screening and diagnosis (45). More recent advances in genomics and related technologies have resulted in the development of a noninvasive prenatal screening (NIPS) test using cell-free fetal DNA sequences isolated from a maternal blood sample. Almost 4-10% of DNA in maternal serum is of fetal origin. Fetal trisomy detection by cfDNA from maternal blood has been done using massively parallel shotgun sequencing (MPSS). By next generation sequencing platforms, millions of amplified genetic fragments can be sequenced in parallel. MPSS detects higher relative amounts of DNA in maternal plasma from the fetal trisomic chromosome compared with reference chromo-somes. Platforms differ according to whether amplified regions throughout the genome, chromosome-specific regions, or single nucleotide polymorphisms (SNPs) are the targets for sequencing (1, 45, 46).

Another approach named digital analysis of selected regions (DANSR) selectively sequences loci only from target chromosomes by including a targeted amplification step. This method represents a considerable increase in sequencing efficiency. Recently, a new method has described selectively the sequences SNPs and ascertain copy number by comparing fetal to maternal SNP ratios between target and reference chromosomes. The use of SNPs may alleviate chromosome- to -chromosome amplification variability; however, the need for a reference chromosome partly negates this advantage (47-50).

**Table 3:** Detection rates and false positive rates of major aneuploidies using NIPT (51, 57, 58)

Chromosome	Detection rate (%) 95 % CI	False positive rate (%) 95 % CI
Trisomy 21	99.2 (98.5–99.6)	0.09 (0.05–0.14)
Trisomy 18	96.3 (94.3–97.9)	0.13 (0.07–0.20)
Trisomy 13	91.0 (85–95.6)	0.13 (0.05–0.26)
Monosomy X	90.3 (85.7–94.2)	0.23 (0.14–0.34)

CI: confidence interval.

Although studies are hopeful and exhibit high sensitivity and specificity with low false- positive rates, there are drawbacks to NIPS. Specificity and sensitivity are not consistent for all chromosomes; this is due to different content of cytosine and guanine nucleotide pairs. False- positive screening results take place and because the sequences derived from NIPS are derived from the placenta, like in chorionic villus sampling (CVS), they may not reflect the true fetal karyotype. Therefore, currently invasive testing is recommended for confirmation of a positive screening test and should remain an option for patients seeking a definitive diagnosis (35, 45, 51).

NIPS for fetal aneuploidy was presented into clinical practice in November 2011. Obstetricians have rapidly accepted this testing, and patients have welcomed this option due to its lack of fetal morbidity and mortality (52). At first, NIPS began as a screen for only trisomy 21 (T21) and was rapidly developed to include other common aneuploidies for chromosomes 13 (T13), 18 (T18), X, and Y (53). 

Notwithstanding improvement in sensitivity, approaches using cfDNA are not diagnostic tests as false positive and false negative results are still generated, although at very low rates than the previous maternal screening tests. A significant source of a discrepant result comes from the fact that the fetal fraction of cfDNA originates pre-dominantly from apoptosis of the trophoblast layer of the chorionic villi and not the fetus. Thus, inva-sive diagnostic testing such as CVS or amnio-centesis, is recommended after a positive cfDNA fetal aneuploidy screening test. Because cfDNA testing is normally presented in the first trimester, CVS is often the choice invasive method applied. If mosaicism is recognized on CVS, confirmatory amniocentesis is recommended (54-56).

Although NIPS is not a diagnostic test, it offers a considerably developed screen for fetal aneuploidy compared to the earlier screening tests that depend on maternal serum markers (Table3). Patients with positive screen results should take suitable genetic counseling to persuade that follow-up testing is necessary before making a decision as to whether or not to continue a pregnancy because of concern over a positive NIPS result. However, patients with negative test results need to know that there is still a chance that their fetus may have a chromosome abnormality due to a false negative result (52).

## Diagnostic methods

Amniocentesis is the most conventional invasive prenatal diagnostic method accepted in the world. Amniocenteses are mostly performed to acquire amniotic fluid for karyotyping from 15 weeks onwards. Amniocentesis performed before 15 weeks of pregnancy is referred to as early amniocentesis. CVS is usually performed between 11 and 13 (13+6) weeks of gestation and includes aspiration or biopsy of placental villi. Amniocentesis and CVS are quite reliable but increase the risk of miscarriage up to 0.5 to 1% compared with the background risk (59-60).

## Treatment

There is no medical cure for DS. However, children with DS would benefit from early medical support and developmental interventions initiation during childhood. Children with DS may benefit from speech therapy, physical therapy and work-related therapy. They may receive special education and assistance in school. Life expectancy for people with DS has improved noticeably in recent decades (61). Nowadays, cardiac surgery, vaccinations, antibiotics, thyroid hormones, leukemia therapies, and anticonvulsive drugs (e.g, vigabatrin) have significantly improved the quality of life of individuals with DS. Actually, life expectancy that was hardly 30 years in the 1960s is now increasing more than 60 years of age (3, 62-63).

X inactivation is the mammalian dosage compensation mechanism that ensures that all cells in males and females have one active X chromoso-me (Xa) for a diploid set of autosomes. This is achieved by silencing one of the two X chromoso-mes in female cells. The X chromosome silencing is effected by Xist non-coding RNA and is associated with chromatin modification (64). Recently, resear-chers have applied this model of transcriptional silencing to the problem of additional gene expre-ssion in DS. In induced pluripotent stem (iPS) cells derived from a patient with DS, the researchers used zinc-finger nucleases to insert inducible X inactive specific transcript (non–protein-encoding) (XIST) into chromosome 21. The mechanism of transcriptional silencing due to the Xist transgene appears to involve covering chromosome 21 with Xist RNA that results in stable modification of heterochromatin. In the iPS cells, induction of the newly inserted transgene resulted in expression of XIST noncoding RNA that coated chromosome 21 and triggered chromosome inactivation (65-66).

## Conclusion

In summary, DS is a birth defect with huge medical and social costs and at this time there is no medical cure for DS. So, it is necessary to screen all pregnant women for DS. NIPS for fetal aneuploidy which was presented into clinical practice since November 2011 has not been yet considered as diagnostic test as false positive and false negative test results are still generated. Thus, invasive diagnostic testing such as CVS or amniocentesis, is recommended after a positive cfDNA fetal aneuploidy screening test.

The described performance of screening for trisomy 21 by the cffDNA test, with a diagnostic rate of more than 99% and false positive rate less than 0.1%, is preferable to other screening methods. Despite the test is obtaining common acceptability, the high cost restricts its application to all patients, identified as such by another traditional first-line method of screening. In the screening with cffDNA testing, the nuchal scan is considered to be the most appropriate first-line method of screening. 

## Conflict of interest

The authors declared no conflict of interest.
